# A Novel Design Principle for the Insect Odorant Receptor

**DOI:** 10.1371/journal.pbio.0040034

**Published:** 2006-01-17

**Authors:** Liza Gross

From flies to lions, smell figures prominently in survival, pointing to food, predators, and mates. For humans, smell plays mostly an aesthetic role, prompting poetic ruminations on evocative aromas and intoxicating bouquets of fine wine. Insects and mammals have evolved common anatomical and physiological features and similar strategies to discriminate among thousands of aromas, using a large repertoire of odorant receptors.

Odorant receptors in mammals belong to a family of signaling proteins called G protein–coupled receptors (GPCRs). Since insects share the GPCR's characteristic seven-transmembrane domain structure (which has an extracellular N-terminus), it has been suggested that insect odorant receptors are a divergent class of GPCRs. But little is known about the signaling pathway activated by the insect receptors. In a new study, Richard Benton, Leslie Vosshall, and colleagues examined the molecular basis of insect olfaction in the fruit fly, Drosophila melanogaster, and show that insects use a “completely different molecular solution to detect odors.”

Odorant receptors are localized to the ciliated tips of olfactory sensory neuron dendrites. In mammals, olfactory sensory neurons express just one odor-specific receptor while insects express these conventional receptors along with a broadly expressed, evolutionarily conserved receptor called odorant receptor 83b (OR83b). It's thought that OR83b collaborates with conventional receptors, based on evidence that odor responses are impaired in *Or83b* mutant fruit flies and that levels of the conventional receptors, such as OR22a/b, drop markedly in OR83b-deficient neurons. [Fig pbio-0040034-g001]

**Figure pbio-0040034-g001:**
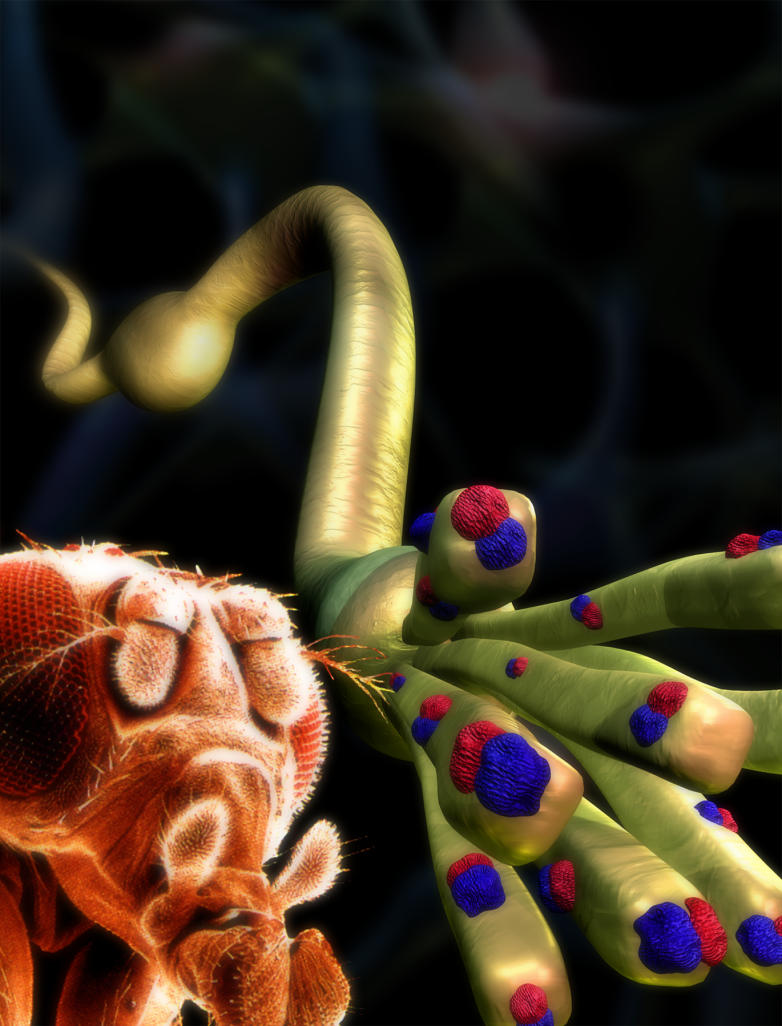
Olfactory receptors localized to the cilia of sensory neurons in Drosophila

With little evidence of OR83b's molecular modus operandi, Benton et al. analyzed the subcellular localization, interactions, and function of OR83b and conventional odorant receptors in Drosophila sensory neurons. By visualizing the localization of OR22a/b in *Or83b* mutant neurons in relation to organelles involved in protein trafficking, the authors found that conventional receptors got stuck in the endoplasmic reticulum (where proteins are synthesized) in the absence of OR83b. OR83b, on the other hand, can reach the cilia on its own, suggesting that OR83b is required to link conventional odorant receptors to the transport machinery. The authors confirmed this by showing that OR83b can promote the localization of odorant receptors to the cilia of neurons that are not normally involved in the detection of odors, converting them into new olfactory neurons. Benton et al. went on to show that OR83b forms protein complexes with conventional odorant receptors, indicating it forms a direct physical link between the transport machinery and the odorant receptor.

Which part of the odorant receptor mediates these interactions? Though previous computational analyses predicted a seven-transmembrane domain in Drosophila odorant receptors, fly sequences resemble no known GPCRs. Benton et al. ran their own computational analysis, comparing fly and mouse odorant receptor sequences, which predicted that fly odorant receptors have seven-transmembrane domains but with an intracellular N-terminus. To experimentally test this surprising prediction, they developed a novel “topology sensor”—using fluorescent proteins designed to sense extracellular versus intracellular domains—and found that the N-terminus of both OR83b and a conventional odorant receptor was indeed located inside the cell, opposite to that of GPCRs. The authors go on to show that conventional odorant receptors interact with OR83b through conserved parts of the proteins that are located inside the cell.

While both insects and mammals transform odors into an internal neural representation, this study reveals that insects use different molecular mechanisms, relying on a novel family of transmembrane proteins that form a complex of two different receptor types: the conventional receptor recognizes odor molecules while OR83b helps to stabilize and transport this receptor to the part of the cell exposed to odors. Since the nature and configuration of the odorant receptor complexes appear unique to insect olfaction, the authors suggest the complexes could prove effective targets against insect vectors of human disease. Future studies can further explore the evolution of this divergence and the molecular basis of odor recognition and olfaction.

